# Eccrine porocarcinoma of the vulva: a case report and review of the literature

**DOI:** 10.1186/s13256-016-1106-1

**Published:** 2016-11-10

**Authors:** Ayako Fujimine-Sato, Masafumi Toyoshima, Shogo Shigeta, Asami Toki, Takashi Kuno, Izumi Sato, Mika Watanabe, Hitoshi Niikura, Nobuo Yaegashi

**Affiliations:** 1 Department of Obstetrics and Gynecology, Tohoku University Graduate School of Medicine, 1-1, Seiryo, Aoba, Sendai, 980-8574 Japan; 2 Department of Pathology, Tohoku University Graduate School of Medicine, 1-1, Seiryo, Aoba, Sendai, 980-8574 Japan; 3 Present address: Department of Obstetrics and Gynecology, Sendai Red Cross Hospital, 2-43-3, Yagiyama-honcho, Taihaku, Sendai, Japan

**Keywords:** Case report, Eccrine gland, Immunohistochemistry, Porocarcinoma, Vulva, Squamous cell carcinoma

## Abstract

**Background:**

Malignant tumors arising from the vulva account for only 0.6 % of all cancers in female patients. The predominant histologic type, representing about 90 % of these malignancies, is squamous cell carcinoma. Eccrine porocarcinoma is a rare malignant tumor arising from sweat glands. The incidence of eccrine porocarcinoma is estimated at 0.005–0.01 % of all cutaneous tumors. To the best of our knowledge, only seven previous cases of vulvar eccrine porocarcinoma have been reported in the English-language literature. We present the case of a patient with eccrine porocarcinoma of the vulva, and we summarize the clinical features of this disease using seven previously reported cases.

**Case presentation:**

A 54-year-old Japanese woman visited a local hospital complaining of fever and left vulvar pain for 2 months. An initial examination revealed a 1 × 1 cm, firm, ulcerative mass in the inner aspect of the left labium minorum. With a preoperative diagnosis of vulvar squamous cell carcinoma, we performed a radical local excision followed by bilateral inguinal lymphadenectomy. Histological examination showed eccrine porocarcinoma, stage IB (T1bN0M0). Radiation therapy with weekly cisplatin administration was then given as adjuvant therapy. One month after treatment was completed, computed tomography revealed multiple metastases in the bilateral lungs and in the sacral bone. The patient received three courses of chemotherapy (paclitaxel and carboplatin) and underwent palliative radiation therapy to the sacrum. She died of her disease 12 months after surgery.

**Conclusions:**

We report the case of a patient with eccrine porocarcinoma of the vulva and summarize the clinical features and the treatment options of eccrine porocarcinoma from a few retrospective case reports. Although eccrine porocarcinoma is a rare disease, clinicians and pathologists should be aware of its clinical and histological features and its biological behavior.

## Background

Malignant tumors arising from the vulva account for 5 % of reproductive organ cancers and 0.6 % of all cancers in female patients [1]. The predominant histologic type is squamous cell carcinoma (SCC), representing about 90 % of all cases. Less common histologic types include melanoma (5–10 % of malignant vulvar cancers) and basal cell carcinoma (approximately 2 %). Eccrine porocarcinoma (EPC) is a very rare malignant tumor; it arises from sweat glands and accounts for an estimated 0.005–0.01 % of all cutaneous tumors [2, 3]. There are two pathways of EPC tumorigenesis: de novo generation and transformation from a benign precursor lesion such as a poroma. EPC should be considered in the differential diagnosis of skin cancer when the tumor is unusually deep and there is no obvious epidermal involvement. To the best of our knowledge, only seven other cases of vulvar eccrine porocarcinoma have been reported in the English-language literature [4–9]. Here, we report a patient with EPC of the vulva. We summarize the clinical features of the disease, using our patient and seven previously reported patients.

## Case presentation

**Fig. 1:**
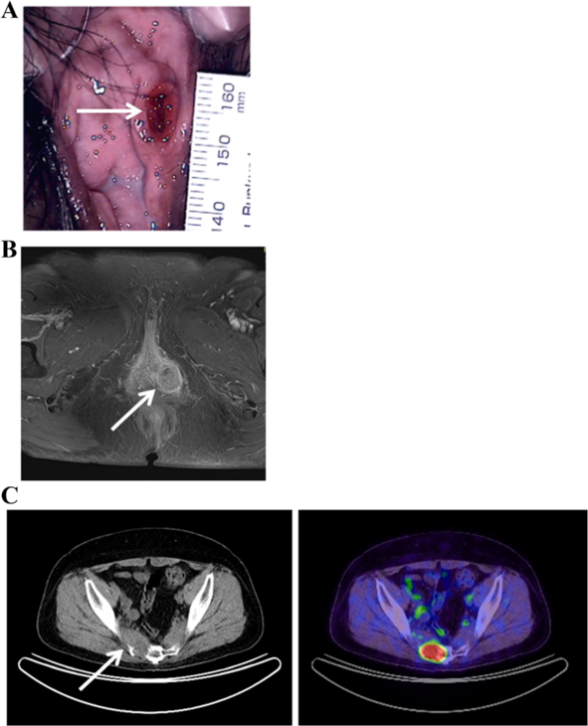
Physical examination and clinical imaging. **a** Ulcerative lesion on the inner side of the left labium minorum (*arrow*). **b** T1-weighted, contrast-enhanced magnetic resonance image revealing a 31 × 24 mm mass of moderate-to-high intensity in the left vulvar region (*arrow*); the mass does not involve the urethra or anus. **c** Positron emission tomography computed tomography revealing tumor metastases to the sacrum (*arrow*)

A 54-year-old gravida 2 para 2 Japanese woman visited her local hospital, complaining of fever and left vulvar pain over a 2-month period. She had a past medical history of appendicitis at 13 years of age, erythema nodosum at 20 years of age, and hypertension diagnosed at 50 years of age. Her menopause occurred at 53 years of age. The initial examination revealed a 10 × 6 mm, firm, ulcerative mass on the inner side of the left labium minorum (Fig. 1a). A vulvar abscess was suspected at the initial visit, but treatment with antibiotics was not effective. SCC was strongly suspected by a subsequent needle biopsy, and she was referred to our hospital for further treatment. Upon admission, her physical examination revealed an ulcerative mass on the left side of the vulva that had increased in size to 31 × 24 mm. Our patient underwent a diagnostic workup that included magnetic resonance imaging, revealing that the primary lesion did not involve the urethra or anus (Fig. 1b). Neither inguinal lymphadenopathy nor distant metastases were seen on computed tomography (CT). With a preoperative diagnosis of vulvar SCC, our patient underwent radical local excision and bilateral inguinal lymphadenectomy. Histopathological examination found eccrine porocarcinoma with a depth of invasion of more than 20 mm; marked vascular invasion was present. The inguinal lymph nodes were negative for metastatic disease, and the surgical margins of the resected lesion were also negative. Our patient was diagnosed with stage IB disease (T1bN0M0; International Federation of Gynecology and Obstetrics 2008). To prevent local recurrence, she underwent adjuvant radiation therapy for the pelvis, bilateral inguinal area, and vulva (50.4 Gy/28 Fr) with concurrent administration of weekly cisplatin (40 mg/m^2^) for five cycles. One month after her treatment was completed, positron emission tomography CT revealed metastases to the sacrum (Fig. 1c) and multiple small nodes in both lungs. She received three further courses of chemotherapy (paclitaxel and carboplatin) and underwent palliative radiation therapy to the sacrum. At 12 months after surgery, our patient died of her disease.

### Histopathologic findings

**Fig. 2:**
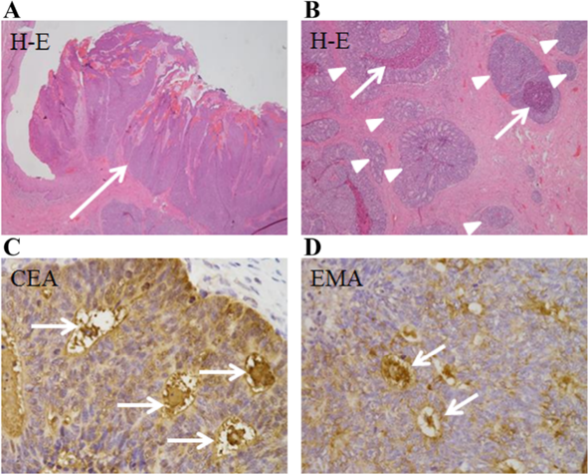
Histopathology and immunohistochemistry of sections of vulva tumor. **a** Tumor cells appearing as solid nests of cells resembling large alveolar glands, broadly connected to the epidermis (*arrow*) (hematoxylin and eosin [H&E] staining; magnification × 40). **b** Tumor cells forming small glandular structures (*arrowheads*); areas of comedo-necrosis are seen (*arrows*) (H&E; magnification × 100). **c** Tumor cells immunopositive for carcinoembryonic antigen at the lumen (*arrows*) (magnification × 400. **d** Tumor cells immunopositive for epithelial membrane antigen at the lumenal rims (*arrows*) (magnification × 400)

Histological examination revealed polygonal tumor cells with variable nuclear atypia and increased mitotic activity. The tumor lesions were broadly connected to the epidermis, but the presence of a direct connection to the squamous epithelium was unclear (Fig. 2a). The tumor cells appeared as solid nests resembling large alveolar glands, without apparent keratinization (Fig. 2b). In addition to the intracytoplasmic luminal and cribriform pattern, necrosis was seen in the center of the nodules, giving the appearance of comedo-necrosis (Fig. 2b). Based on these findings on hematoxylin and eosin-stained sections, the differential diagnosis included SCC, adenosquamous cell carcinoma, adenocarcinoma, sweat gland carcinoma, and mammary-type ductal adenocarcinoma. Additional immunohistochemical examinations were performed (Table 1). The tumor was strongly positive for CAM5.2, partially positive for 34βE12, focally positive for carcinoembryonic antigen (CEA) (Fig. 2c), and negative for cancer antigen 125 expression. The tumor was strongly positive for p16 expression, and almost negative for the squamous cell markers p63 and p40. The neuroendocrine markers chromogranin A and synaptophysin were partially positive. The tumor was negative for the apocrine marker BRST-2 (also known as gross cystic disease fluid protein-15); estrogen, progesterone, and androgen receptors; human epidermal growth factor receptor 2 (HER2); p53; and mucin 1, cell surface associated (MUC-1) expression. Epithelial-membrane antigen (EMA) expression was positive at the luminal rims (Fig. 2d). The tumor was focally positive for S-100 and positive for Ber-EP4 expression. These findings were consistent with the diagnosis of vulvar porocarcinoma.

**Table 1:** Summary of immunohistochemical examinations

Antibody	CAM5.2	34βE12	CEA	CA125	p16	p63	p40	Chromogranin A	Synaptophysin	BRST-2
Staining	++	+	+	-	++	-	-	+	+	-
Antibody	ER	PR	AR	HER2	p53	MUC-1	EMA	S-100	Ber-EP4	
Staining	-	-	-	-	-	-	+	+	+	

*Abbreviations*: *CAM5.2* Cytokeratin CAM5.2, *34βE12* Cytokeratin 34βE12, *CEA* carcinoembryonic antigen, *CA125* cancer antigen 125, *ER* estrogen receptor, *PR* progesterone receptor, *AR* androgen receptor, *HER2* human epidermal growth factor receptor 2, *MUC-1* mucin 1, cell surface associated, *EMA* epithelial membrane antigen, *S-100* S-100 protein

## Discussion

Primary adenocarcinoma arising from the eccrine sweat glands is very rare, representing approximately 0.005 % of epithelial cutaneous neoplasms [3]; EPC is the most common variant of this type of malignancy. Macroscopically, EPC can appear as a nodule, a polypoid ulcerating lesion, or a plaque with an infiltrative or erosive pattern. The lower extremities and the head and neck are the most frequent sites of EPC origin [10, 11]. It is very rare for the lesion to arise in the vulva: only seven cases of invasive vulvar EPC have been reported in the English-language literature (Table 2). The mean age at diagnosis in these seven patients was 61 years (range, 32–80 years). Four patients had a favorable outcome with surgical treatment alone, but the other four, including our patient, had recurrent or metastatic disease in spite of multidisciplinary treatment.

**Table 2:** Summary of eight reported cases with eccrine porocarcinoma of the vulva

Age (years)	Location, (size:cm)	Initial treatment	Additional treatment	Outcome	Author
80	Perineum, labium, (N/A)	Excision and postoperative radiation	Radical vulvectomy for multiple recurrent region	Death due to unrelated disease	Wick, 1985 [4]
75	Labium majus, (2 × 3)	Radical hemivulvectomy and lymph node dissection, postoperative radiation	None	No evidence of disease	Katsanis, 1996 [5]
88	Labium majus, (3 × 2)	Local excision	None	No evidence of disease	Stephen,1998 [6]
32	Mons pubis, labium majus, (4.5)	Chemotherapy and radiation	None	Early extensive metastasis	Liegl, 2005 [7]
60	Labium majus, (3)	Chemotherapy	Excision and postoperative radiation for recurrent region	Live with recurrent tumor	Liegl, 2005 [7]
54	Perineum, (10)	Surgery	None	No evidence of disease	Iannicelli, 2008 [8]
48	Labium majus, (5 × 4.5 × 2.5)	Excision and lymph node dissection	None	No evidence of disease	Adegboyega, 2011 [9]
54	Vaginal vestible, (3 × 2)	Excision and lymph node dissection, postoperative concurrent chemoradiotherapy	Chemotherapy for the lung metastasis, radiation for the sacral bone metastasis	Early extensive metastasis	Present case

*N/A* not available

Local and regional recurrence rates of EPC are reportedly approximately 20 % [11]. The distant metastasis rate is reportedly 12 %, and, more importantly, the mortality rate of metastatic EPC ranges from 75 to 80 % [10]. EPC usually has a poor prognosis, regardless of aggressive therapy, especially when it affects younger patients [3, 7]. The following histological factors are associated with aggressive behavior in EPC: tumor depth greater than 7 mm, lymphovascular invasion, and greater than 14 mitotic figures per 10 high-power fields [10]. Our patient’s tumor met all three criteria. EPC has been classified into three subtypes: infiltrative, pushing, and pagetoid, based on the infiltrative pattern at the margins [10, 12]. The infiltrative and pagetoid patterns are predictive of local recurrence [10]. Belin et al. reported that four of ten patients with infiltrative and two of two with pagetoid EPC developed recurrence, whereas none of the seven patients with a pushing pattern experienced recurrence. Based on these findings, they propose a surgical decisional algorithm based on the EPC histological subtype [12].

A histopathological misdiagnosis of EPC might result in inappropriate treatment and an unfavorable outcome. Unfortunately, the precise diagnosis of EPC lesions can be difficult because of their rareness and diverse histological features that are somewhat similar to those of other skin neoplasms. EPC has heterogenous histology that includes squamous cells, mucinous cells, clear cells, pigmented cells, and spindle cells [9]. Morphologically varied tumor cells may contribute to the relatively high rates of misdiagnosis, particularly from small biopsy specimens. A report by Belin et al. described 37 % of patients with skin EPC receiving the wrong diagnosis based on initial histological evaluation [12]. In the same report, five of 24 EPC lesions were misdiagnosed as SCC [12], indicating that the initial misdiagnosis of the biopsy in our patient was not unusual.

Most patients with EPC are treated with a surgical procedure that includes wide local excision and evaluation of margins by frozen section. The routine dissection of regional lymph nodes remains controversial, although lymphadenectomy is indicated for patients with clinically enlarged nodes. Radiation therapy for EPC is ineffective for the control of recurrence or metastatic disease [3, 5]. In addition, metastatic EPC has proven resistant to many cytotoxic agents [13]. Combination chemotherapy regimens obtain only short remission times and frequently have severe adverse effects [14]. Therefore, chemotherapy has only been used sporadically. Currently, no effective treatment for advanced or relapsed EPC has been found; more information is needed.

## Conclusions

In conclusion, we report a patient with EPC of the vulva and summarize the clinical features and the treatment options using the few available retrospective case reports. The diagnostic assessment of a biopsy specimen, which may not contain definitive histological features, can lead to a misdiagnosis of SCC. Although EPC is a rare disease, clinicians and pathologists should be aware of its clinical and histological features and its biological behavior.

## Abbreviations

CEA: carcinoembryonic antigen
CT: computed tomography
EMA: epithelial-membrane antigen
EPC: eccrine porocarcinoma
HER2: human epidermal growth factor receptor 2
MUC1: mucin 1, cell-surface associated
SCC: squamous cell carcinoma
